# Ginsenoside Rg1 inhibits nucleus pulposus cell apoptosis, inflammation and extracellular matrix degradation via the YAP1/TAZ pathway in rats with intervertebral disc degeneration

**DOI:** 10.1186/s13018-022-03443-4

**Published:** 2022-12-20

**Authors:** Yong-hua Yang, Xiao-peng Gu, Hong Hu, Bin Hu, Xiang-lian Wan, Zhi-ping Gu, Shao-jin Zhong

**Affiliations:** 1 The Third Department of Orthopedics, Jiujiang First People’s Hospital, Jiujiang, 332000 Jiangxi China; 2 Department of Orthopaedics, Zhoushan Guhechuan Bone Injury Hospital, Zhoushan,, 316101 Zhejiang China; 3 The Nursing Department, Jiujiang First People’s Hospital, Jiujiang, 332000 Jiangxi China; 4 Pharmaceutical Department, Orthopedics and Diabetes Hospital in Haikou, Shanghai Sixth People’s Hospital, Haikou, 570311 Hainan China; 5 Pharmaceutical Department, Affiliated Haikou Hospital of Xiangya Medical College, Central South University, Haikou, 570208 Hainan China

**Keywords:** Intervertebral disc degeneration, Nucleus pulposus cells, Ginsenoside Rg1, YAP1/TAZ signaling pathway

## Abstract

**Purpose:**

Intervertebral disc degeneration (IDD) is one of the main causes of low back pain, which not only affects patients’ life quality, but also places a great burden on the public health system. Recently, ginsenoside Rg1 has been found to act in IDD; however, the mechanism is still unclear. The purpose of this study is to explore the function of ginsenoside Rg1 and its molecular mechanism in IDD.

**Methods:**

The rat model of IDD and nucleus pulposus (NP) experimental groups treated with ginsenoside Rg1 was constructed for investing the role of ginsenoside Rg1 in IDD rats. In the in vitro and in vivo study, the histological morphological changes, motor threshold (MT), inflammatory factors, oxidative stress, apoptosis and expression of the YAP1/TAZ signaling pathway-related proteins of the intervertebral discs (IVD) were measured by histological staining, mechanical and thermal stimulation, ELISA, qRT-PCR, flow cytometry, and western blot, respectively.

**Results:**

Ginsenoside Rg1 significantly increased the threshold for mechanical and thermal stimulation and alleviated histological changes in IDD rats. Ginsenoside Rg1 had a significant inhibitory effect on the secretion level of inflammatory factors, redox activity, extracellular matrix (ECM) degradation in IVD tissue and NP cells, and apoptosis in NP cells. Further investigation revealed that ginsenoside Rg1 significantly inhibited the expression of YAP1/TAZ signaling pathway-related proteins. Additionally, the above inhibitory effect of ginsenoside Rg1 on IDD progression was concentration-dependent, that is, the highest concentration of ginsenoside Rg1 was most effective.

**Conclusion:**

Ginsenoside Rg1 inhibits IDD progression by suppressing the activation of YAP1/TAZ signaling pathway. This means that ginsenoside Rg1 has the potential to treat IDD.

## Introduction

Low back pain (LBP) is the main cause of disability, and approximately 84% of people suffer from LBP worldwide [1]. According to statistics, 10% LBP patients develop into chronic disability, resulting in reduced quality of life and bringing a heavy economic burden to families and society [2]. The causes of LBP are very complex, and intervertebral disc degeneration (IDD) has been reported as a main cause leading to approximately 40% of all LBP patients [3]. The intervertebral disc (IVD) is located between the adjacent vertebral bodies, which provides load support, flexibility, energy storage in the spine [4]. IVD is composed of a gel-like nucleus pulposus (NP) surrounded by a fibrocartilaginous annulus fibrosus (AF). NP cells form a complex extracellular matrix (ECM) by secreting collagen type II (COL II) and proteoglycans [5]. IDD originates in the NP and is characterized by excessive apoptosis of NP cells and degradation of ECM [6, 7]. There are many inducing factors for IDD, including genetics, ageing and gender. At present, the clinical interventions for IDD mainly include conservative drug therapy and surgery [8]. However, the existing treatment options can not solve the problem fundamentally, but only temporarily alleviate the pain. Therefore, it is particularly important to find new drugs or treatments that bring disease to cure LBP.

Ginseng is one of the most widely used natural medicines in the world and is also a focus in herbal medicine research due to its diverse pharmacological activities [9]. Ginsenoside Rg1, a tetracyclic triterpenoid derivative, is the main active component of ginseng [10]. There is evidence to suggest that ginsenoside Rg1, which has been used in clinical practice, has a wide range of pharmacological effects on the central nervous system, cardiovascular system, digestive system and endocrine system [11]. For example, ginsenoside Rg1 can attenuate brain water content, promote neurogenesis, resist apoptosis, boost energy and cerebral circulation [12]. Xiao et al. [13] have confirmed that ginsenoside Rg1 ameliorates palmitic acid-induced hepatic steatosis and inflammation via regulating AMPK/NF-*κ*B signaling pathway. Xiang et al. [14] have found that ginsenoside Rg1 can promote the proliferation of neural stem cells, antagonize the senescence of neural stem cells and delay brain ageing by inhibiting the activation of the Wnt/*β*-catenin pathway. Qin et al. [15] have reported that ginsenoside Rg1 enhances mitochondrial biogenesis by increasing PGC-1*α*, complex III and complex IV expression, thus attenuating symptoms of cardiac hypertrophy and hypertension and decreasing oxidative stress and inflammation. Collectively, it can be seen that ginsenoside Rg1 can alleviate apoptosis, senescence, inflammation, and oxidative stress. Further, Yu et al. [16] have demonstrated that ginsenoside Rg1 promotes NP cell proliferation to improve IDD by regulating the Wnt/*β*-catenin pathway. However, the study by Yu et al. can not comprehensively reveal the mechanism of ginsenoside Rg1 on IDD. Given this lack, this study investigated the effect and mechanism of ginsenoside Rg1 on IDD to provide new methods for treating this disease.

## Materials and methods

### Animal models

All experiments were approved by the Ethics Committee of Jiujiang First People’s Hospital. Fifty 8-week-old female SD rats weighing 220 ± 30 g were selected in the study (5 groups, 10 rats/group). The specific steps of constructing a rat model of IDD were as follows [17]. First, the rats were anaesthetized with intraperitoneal injection of 2% (wt/vol) pentobarbital, and the depth of anaesthesia was determined by a toe-pinch test. Then, their tails and limbs were fixed, and the coccygeal IVD (Co7/8) was positioned by digital palpation and confirmed by radiography. After that, a 20-gauge needle was inserted horizontally in the AF of Co7/8, and traveled through the NP to reach the contralateral AF. After full penetration, the needle was rotated 360° twice and held for 30 s. The depth of needle penetration was controlled by the resistance of the contralateral AF.

Ginsenoside Rg1 was purchased from Baiaolaibo Technology Co., Ltd (Beijing, China). The rats without any treatment were set as the normal group; the rat models were divided into 4 groups and intragastrically administrated for 4 weeks: IDD group (same amount of saline), L-Rg1 group (20 mg/kg/d Rg1), M-Rg1 group (40 mg/kg/d Rg1), and H-Rg1 group (80 mg/kg/d Rg1). Finally, the rats were euthanized, followed by IVD tissue collection.

### Isolation, culture and treatment of nucleus pulposus cells

The NP was separated from the AF of IVD from the normal group and IDD group under aseptic conditions. Subsequently, the NP tissues were washed with sterile PBS 3 times and cut into small pieces of about 1 mm^3^_._ The pieces were digested with 0.5% (w/v) collagenase type II at 37 °C for 2 h. Next, the digested tissues were cultured in DMEM/F-12 (11,320,033, Gibco) supplemented with 15% foetal bovine serum (FBS) and antibiotics (1% penicillin and streptomycin) in an incubator maintained at 37 °C with 5% CO_2_. The culture medium was changed once every 3 days. For cells with good growth status, 0.25% trypsin (R001100, Gibco) was used. The cells passaged to the third generation were used for in vitro experiments. The isolation protocol of rat NP cells mentioned above referred to the method introduced by Liu et al. [18]. NP cells isolated from the NP tissues of rats in the Normal group were named normal group; similarly, we obtained NP cells named IDD, L-Rg1 (25 µmol/L), M-Rg1 (50 µmol/L) and H-Rg1 (100 µmol/L) groups. The cells in the H-Rg1 group that were additionally treated with YAP1 were called H-Rg1 + YAP1 group.

### Mechanical withdrawal threshold

The rats were placed on a transparent plexiglass box with a wire floor grid. After the rats habituated for 30 min, their hind paw of the right side (affected side) was stimulated with Von Frey filament of varying forces (Danmic Global, USA) were applied. The force was slowly applied until the rats lifted or licked the paw, and this force was the mechanical stimulation threshold. The evaluation was carried out and repeated 4 times on 0–4 days after surgery, and the average of the 4 times was used for the final statistical analysis. The interval between stimulations at the same site was 3 min to avoid the influence of the previous stimulation on the subsequent stimulation.

### Thermal withdrawal threshold

The rats in each group were placed in a plexiglass box and habituated for 30 min. Then, with an ambient temperature controlled at 25 ± 1 °C, the hind paw of the right side (affected side) was stimulated by 390G analgesimeter (IITC, USA). The time from the start of thermal stimulation to withdrawal response was recorded as the thermal stimulation latency. Thermal stimulation was limited to 60 s per stimulation. On 0–4 days after surgery, the assessment carried out and repeated 4 times, and the average value of 4 times was used for the final statistical analysis. The interval between stimulations at the same site was 3 min to avoid the influence of the previous stimulation on the subsequent stimulation.

### Safranin O-fast green (SO) staining

The first step was the preparation of IVD tissue sections. The IVD tissues of rats in each group were excised, and the soft tissues were removed with IVD tissue integrity maintained during this process. The IVD tissue was then fixed in neutral formaldehyde for 20 min, decalcified in 15% ethylenediaminetetraacetic acid (EDTA) solution, dehydrated with gradient ethanol. After that, the tissues were embedded whole in paraffin, and sliced into the sagittal Sects. (6 μm thick). The second step was the staining procedure. After deparaffinization, the sections were stained with hematoxylin for 7 min followed by 10 min rinsing with tap water, stained with 0.5% fast green for 8 min followed by 15 s rinsing with 1% acetic acid, and finally stained with 0.1% safranin O for 5 min followed by rinsing with 95% ethanol, absolute ethanol, and xylene for 2 min each. The slices were mounted with neural resin after they were dried. Finally, a microscope was utilized to observe the morphology of IVD tissues and collect images, and the histological grades were assessed by staining.

### Hematoxylin–eosin (HE) staining

The slide preparation process was the same as in Sect. “Safranin O-fast green (SO) staining”. Then, the embedded sections were deparaffinized, stained with hematoxylin for 5 min at ambient temperature and differentiated in 1% hydrochloric acid alcohol. After this, the sections bluing in 1% ammonia water were then rinsed with tap water, stained in eosin solution for 30 s. Upon completion of staining, the samples were dehydrated with ethanol, cleared with xylene and mounted with neutral resin after drying. Finally, a microscope was utilized to observe the morphology of IVD tissues and collect images, and the histological grades were assessed by staining.

### Enzyme linked immunosorbent assay (ELISA)

First, 50 mg of NP tissues collected from each group was placed in a homogenization tube and added 1 mL of precooled PBS buffer. The tissues were then homogenized and broken in a low-temperature homogenizer. After the completion of homogenization, the homogenization tube was centrifuged at 12,000 g for 10 min at 4 °C to remove cell debris. The supernatant was dispensed into new centrifuge tubes; part of the supernatant was stored at −80 ℃, while the other part was detected for the expression of necrosis factor *α* (TNF-*α*), interleukin 6 (IL-6), interleukin 1*β* (IL-1*β*), and prostaglandin E2 (PGE2) by using the ELISA detection kit (Nanjing Jiancheng Bioengineering Institute, China).

### Biochemical test

The homogenized tissue supernatant obtained in Sect. “Enzyme linked immunosorbent assay (ELISA)” and cell culture supernatant with cell debris removed by centrifugation were taken. The activities of reactive oxygen species (ROS) and malondialdehyde (MDA) in NP tissue and cell supernatant were detected by automatic biochemical analyser.

### Flow cytometry

The cells in each group were digested into centrifuge tubes using trypsin and rinsed twice with pre-chilled sterile PBS. The cell concentration was adjusted to 5 × 10^5^ cells/mL. Then, 200 μL of cells suspension was taken and added 10 μL of Annexin V-FITC and 10 μL of 20 mg/L phycoerythrin (PE) solution. After that, the cells were incubated for 10 min in the dark at ambient temperature. On completion of incubation, 500 μL of PBS was added for rinsing and then cell apoptosis was examined by flow cytometer.

### qRT-PCR

Total RNA was extracted from NP tissues and NP cells in each group using the TRizol method. Subsequently, the concentration and purity of RNA were determined by NanoDrop, and cDNA was prepared according to random primer reverse transcription kit (Thermo, USA). The expression levels of MMP13, ADAMTS-5, collagen II, and ACAN mRNA were detected according to the instructions of SYBR GREEN kit (TaKaRa, Japan), and GAPDH was used as the internal reference. The relative expression of the product was calculated by the 2^−ΔΔCt^ formula. The sequences of primer are shown in Table 1.

**Table 1:** qRT-PCR primer sequences

Gene name	Sequences (5′ to 3′)
MMP13	F: 5′-TGAAGTAGGACTGGGCAGAGA-3′
	R: 5′-TTTGGGTCAGGTGTCCACTC-3′
ADAMTS-5	F: 5′-CTGCCTTCAAGGCAAATGTGTGG-3′
	R: 5′-CAATGGCGGTAGGCAAACTGCA-3′
COL II	F: 5′-CCTGGCAAAGATGGTGAGACAG-3′
	R: 5′-CCTGGTTTTCCACCTTCACCTG-3′
ACAN	F: 5′-CAGGCTATGAGCAGTGTGATGC-3′
	R: 5′-GCTGCTGTCTTTGTCACCCACA-3′
GAPDH	F: 5′-GTCTCCTCTGACTTCAACAGCG-3′
	R: 5′-ACCACCCTGTTGCTGTAGCCAA-3′

### Western blot

NP tissues and cells were collected, followed by extraction of the total protein of cells by cell lysate (Beyotime, P0013B). Then, the BCA protein assay kit (Beyotime, P0010) was employed to determine the protein concentration. Subsequently, 20 μg of protein with 5 × loading buffer was boiled for denaturation. The proteins were isolated by sodium dodecyl sulphate–polyacrylamide gel electrophoresis (SDS-PAGE) and then transferred onto polyvinylidene fluoride (PVDF) membranes. Afterwards, the membranes were washed with TBST solution for 3 times, 10 min each time, and blocked by 5% skim milk powder for 2 h. On completion of the blocking step, the membranes were incubated with the appropriate primary antibodies (YAP1, Cell Signaling Technology, D8H1X; TAZ, Cell Signaling Technology, V386; TEAD1, Abcam, ab133533; CTGF, Abcam, ab6992; GAPDH, Cell Signaling Technology, D16H11) at 4 °C overnight. The next day, the membranes were incubated with horseradish peroxidase-labeled anti-rabbit IgG (ZSGB-Bio, ZB2301) for 1 h at ambient temperature after washing the membranes with TBST solution for 3 times, 10 min each time. After incubation, the membranes were rinsed with TBST solution three more times, each time for 10 min. Then, enhanced ECL chemiluminescence reagent was used to visualize the proteins and images were captured using the gel imaging system. The grey values of bands were quantified using the Image J analysis software, and the relative protein expression was calculated using GAPDH as an internal reference.

### Statistical analysis

One-way analysis of variance (ANOVA) was processed using SPSS 26.0. The measurement data were presented as mean ± standard deviation. When *p* < 0.05, the results were considered statistically significant.

## Results

### Effects of ginsenoside Rg1 on motor function in rats with intervertebral disc degeneration

**Fig. 1:**
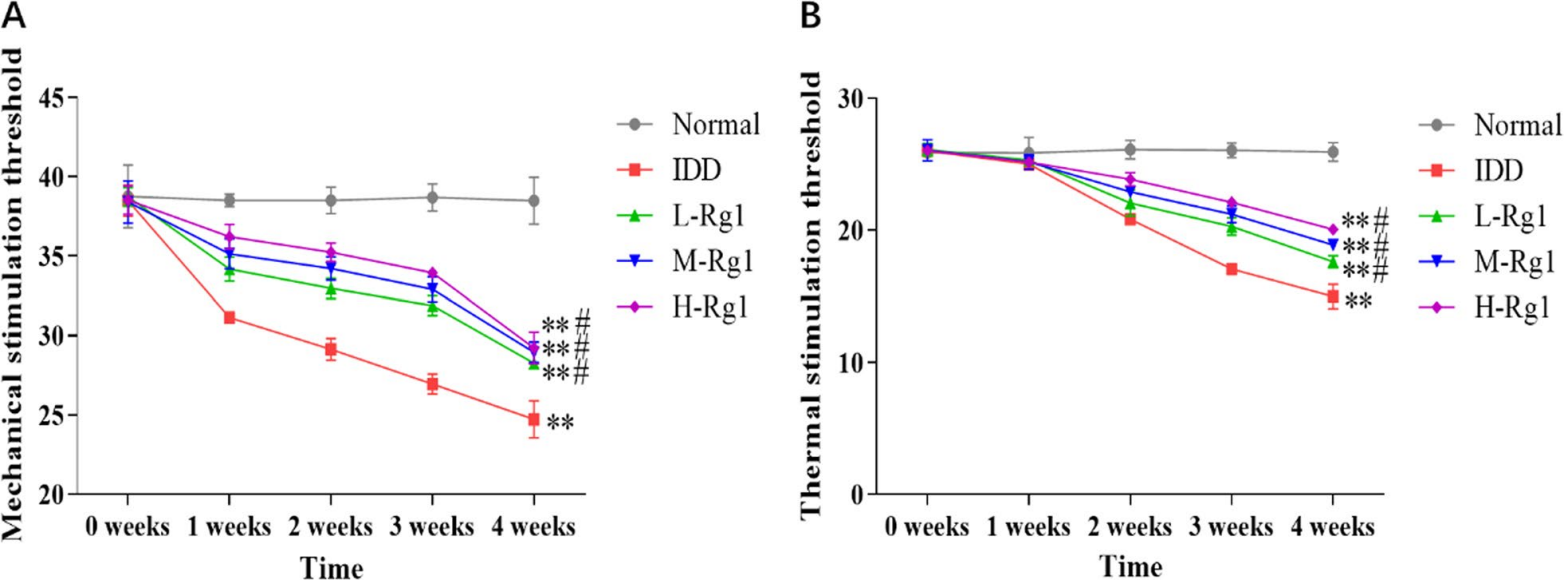
Effects of ginsenoside Rg1 on behaviour in rats with intervertebral disc degeneration. **A**: Mechanical stimulation threshold of rats in each group; **B**: Thermal stimulation threshold of rats in each group. **p < 0.01 versus Normal group; ^#^*p* < 0.05 versus IDD group

At first, we observed the effect of ginsenoside Rg1 on motor function in IDD rats. Compared with the normal group, the mechanical stimulation threshold was markedly decreased in the IDD group; this threshold of IDD rats was considerably increased in the Rg1-treated groups in a dose-dependent manner. But at 4 weeks after surgery, there was no significant difference in the mechanical stimulation threshold among different concentrations of ginsenoside Rg1 (Fig. 1A). Additionally, the threshold for thermal stimulation in IDD rats was significantly lower than in normal rats; however, ginsenoside Rg1 significantly up-regulated this threshold of IDD rats in a dose-dependent manner (Fig. 1B).

### Effect of ginsenoside Rg1 on pathology of nucleus pulposus tissues in rats with intervertebral disc degeneration

**Fig. 2:**
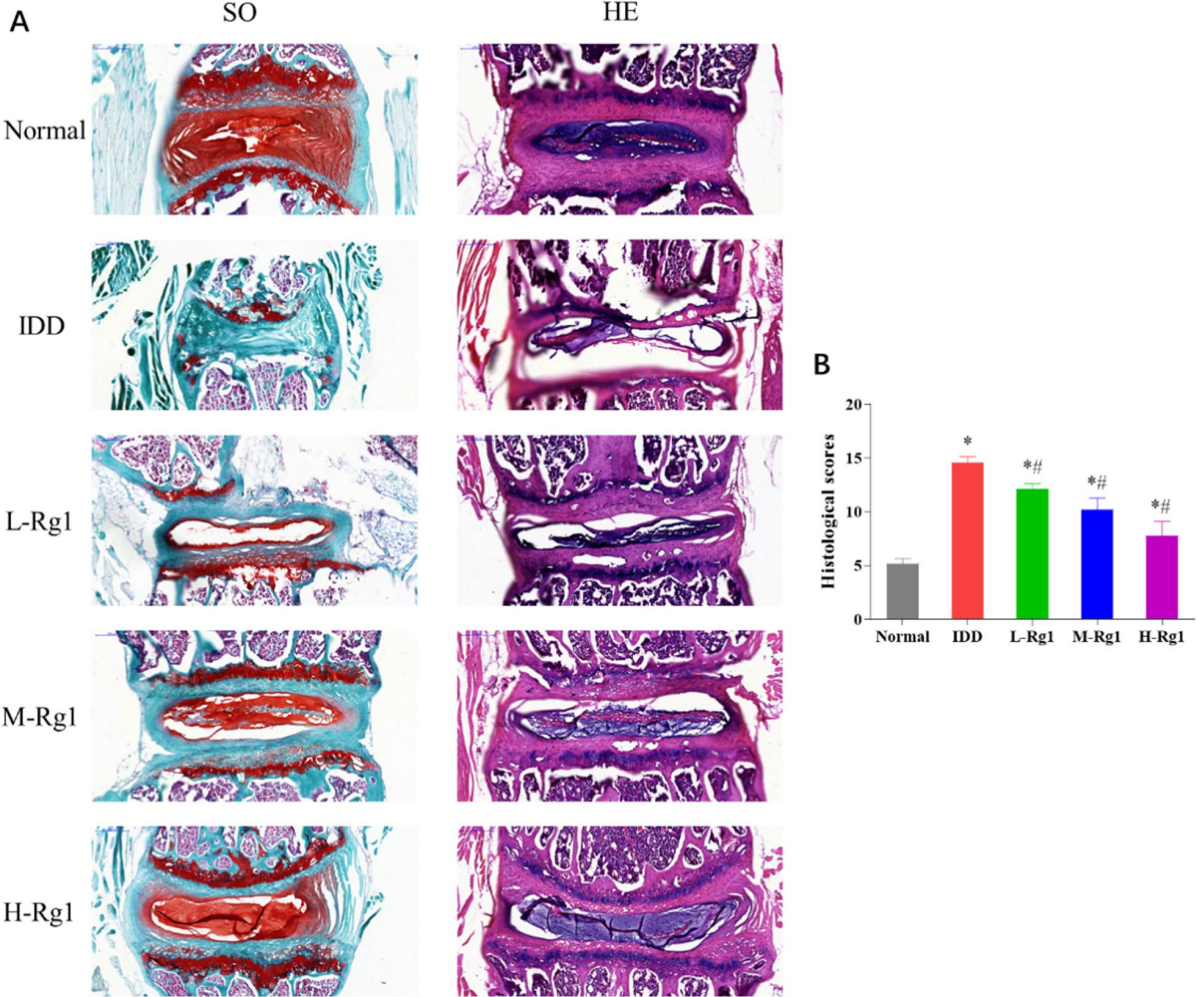
Effect of ginsenoside Rg1 on pathology of nucleus pulposus tissues in rats with intervertebral disc degeneration. **A**: Safranin O-Fast green staining or HE staining results of lumbar intervertebral disc tissue; **B**: Staining-based histological grade of lumbar intervertebral disc tissue in each group, *p < 0.05 versus Normal group; ^#^*p* < 0.05 versus IDD group

To visually observe the effect of ginsenoside Rg1 on the progression of IDD, we performed histological staining analysis of IVD of rats in each group. The results of Safranin O-Fast green staining and HE staining showed that the IVD tissues of IDD rats presented with varying degrees of loss in cartilage matrix and varying levels of damage of cartilage tissue. Additionally, compared with the normal group, IDD rats had decreased size of NP cells, and the boundary between the NP and AF was interrupted. Also, IDD caused nuclear cells to gather and form clusters, and an internal ring formed in the collagen layer disorder of AF. However, administration of ginsenoside Rg1 achieved varying degrees of improvement of IDD and ginsenoside Rg1 concentration was positively correlated with the improvement (Fig. 2A).

The histological scores of the IDD group were considerably increased compared with the normal group; however, the scores of the L-Rg1, M-Rg1, and H-Rg1 groups were significantly declined compared with the IDD rats (Fig. 2B). The data demonstrated that ginsenoside Rg1 could significantly alleviate the progression of IDD-caused injury.

### Ginsenoside Rg1 inhibits the inflammatory response and oxidative stress levels in rats with intervertebral disc degeneration

**Fig. 3:**
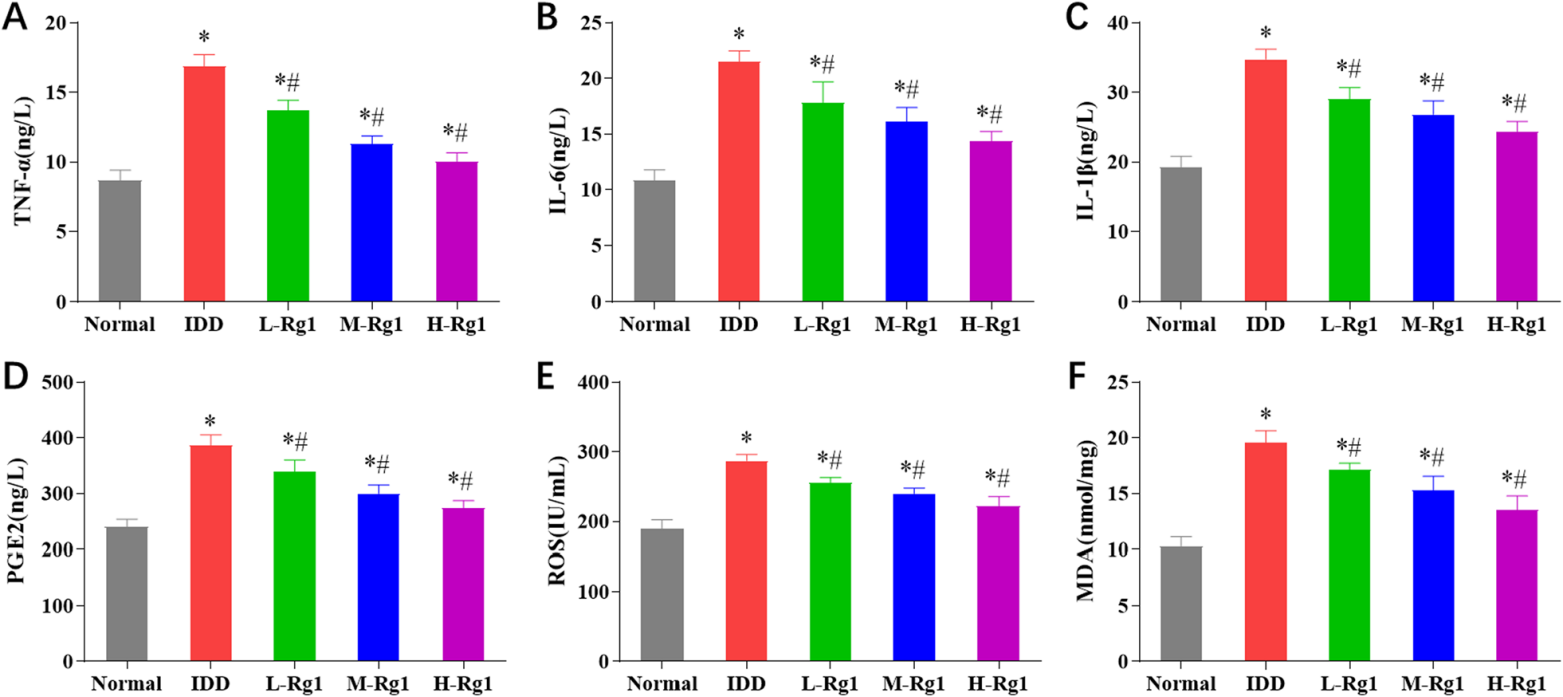
Ginsenoside Rg1 inhibits the inflammatory response and oxidative stress response in rats with intervertebral disc degeneration. **A**–**F**: Levels of TNF-*α* (**A**), IL-6 (**B**), IL-1*β* (**C**), PGE2 (**D**), ROS activity (**E**) and MDA activity (**F**) in NP tissue of rats in each group, *p < 0.05 versus Normal group; ^#^*p* < 0.05 versus IDD group

IDD is characterized by increased levels of the proinflammatory cytokines TNF-*α*, IL-6, IL-1*β*, PGE2 secreted by the IVD cells [19], and strong oxidative stress response promotes NP cell senescence and death to change IVD structure and cause clinical responses. Therefore, we examined the secretion levels of inflammatory factors and ROS levels in the NP tissues to further determine whether ginsenoside Rg1 could reduce the inflammatory response and oxidative stress response in IDD rats. According to the results, the NP tissues of IDD rats showed increased levels of TNF-*α*, IL-6, IL-1*β*, and PGE2, and increased activities of ROS and MDA compared with the normal group; however, ginsenoside Rg1 could significantly decrease such increases in a dose-dependent manner (Fig. 3A–F). Collectively, ginsenoside Rg1 could significantly reduce inflammatory and oxidative stress levels in IDD rats.

### Effect of ginsenoside Rg1 on extracellular matrix degradation in nucleus pulposus tissues in rats with intervertebral disc degeneration

**Fig. 4:**
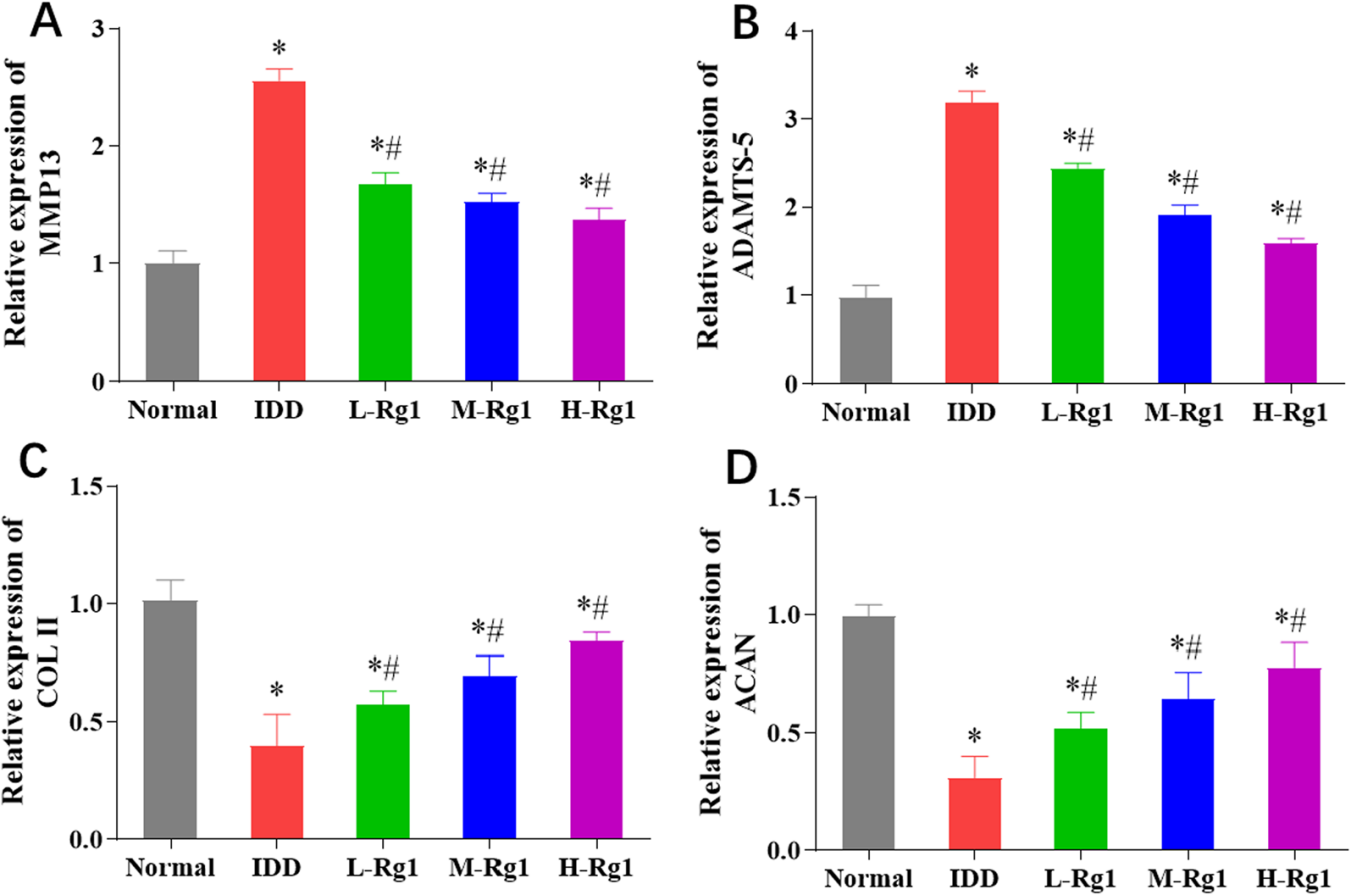
Effect of ginsenoside Rg1 on extracellular matrix degradation in nucleus pulposus tissues in rats with intervertebral disc degeneration. **A**–**D**: mRNA expression level of MMP13 (**A**), ADAMTS-5 (**B**), COL II (**C**) and ACAN (**D**) in NP tissue of rats in each group, *p < 0.05 versus Normal group; ^#^*p* < 0.05 versus IDD group

Matrix metalloproteinases (MMP) and ADAMTS (A Disintegrin and Metalloprotease domains, with thrombospondin motifs) are two major matrix-degrading enzymes involved in IDD; these enzymes promote the degradation of ECM molecules, such as aggrecan (ACAN) and collagen II (COL II) to further induce inflammation [19]. Therefore, in this section, we discussed the effect of ginsenoside Rg1 on ECM degradation-related genes. QRT-PCR results revealed that the expression of MMP13 and ADAMTS-5 was considerably increased while the expression of COL II and ACAN was notably reduced in the NP tissue of the IDD group in comparison with the normal group. By contrast, ginsenoside Rg1 led to significant decreases in MMP13 and ADAMTS-5 expression and increases in COL II and ACAN expression in the NP tissue in a concentration-dependent manner (Fig. 4A–D).

### Ginsenoside Rg1 suppresses the apoptosis rate of nucleus pulposus cells in rats with intervertebral disc degeneration

**Fig. 5:**
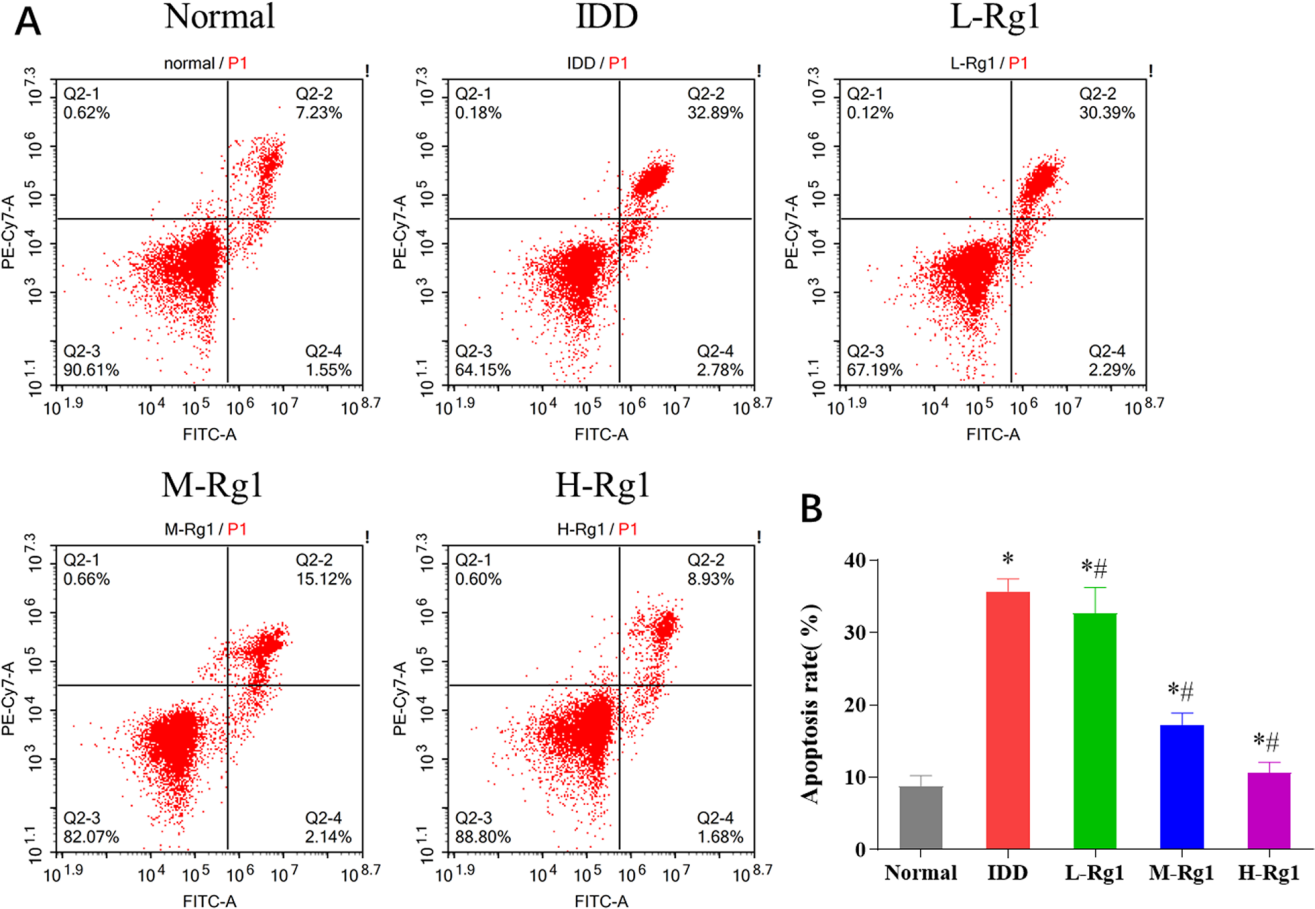
Ginsenoside Rg1 suppresses the apoptosis rate of nucleus pulposus cells in rats with intervertebral disc degeneration. **A**: Apoptosis of NP cells verified by flow cytometry. **B**: Statistical quantification of apoptotic rate in each group, and the data are mean of triplicate measurements ± SD, *p < 0.05 versus Normal group; ^#^*p* < 0.05 versus IDD group

NP cell apoptosis is one of the main reasons for the decrease in NP cell number. To further investigate the effect of ginsenoside Rg1 on the apoptosis of NP cells, we detected the apoptosis of NP cells in each group by flow cytometry. The results suggested that the apoptotic rate of NP cells in the IDD group was remarkably up-regulated compared to the normal group; the apoptotic rate of cells in the L-Rg1, M-Rg1, and H-Rg1 groups was remarkably down-regulated in a concentration-dependent manner compared to the IDD group (Fig. 5A, B). The above results showed that ginsenoside Rg1 could inhibit the apoptosis of NP cells in IDD rats, and the number of apoptosis of NP cells in IDD rats was significantly decreased with the increase in ginsenoside Rg1 concentration.

### Ginsenoside Rg1 inhibits the inflammatory response, oxidative stress and extracellular matrix degradation of nucleus pulposus cells in rats with intervertebral disc degeneration

**Fig. 6:**
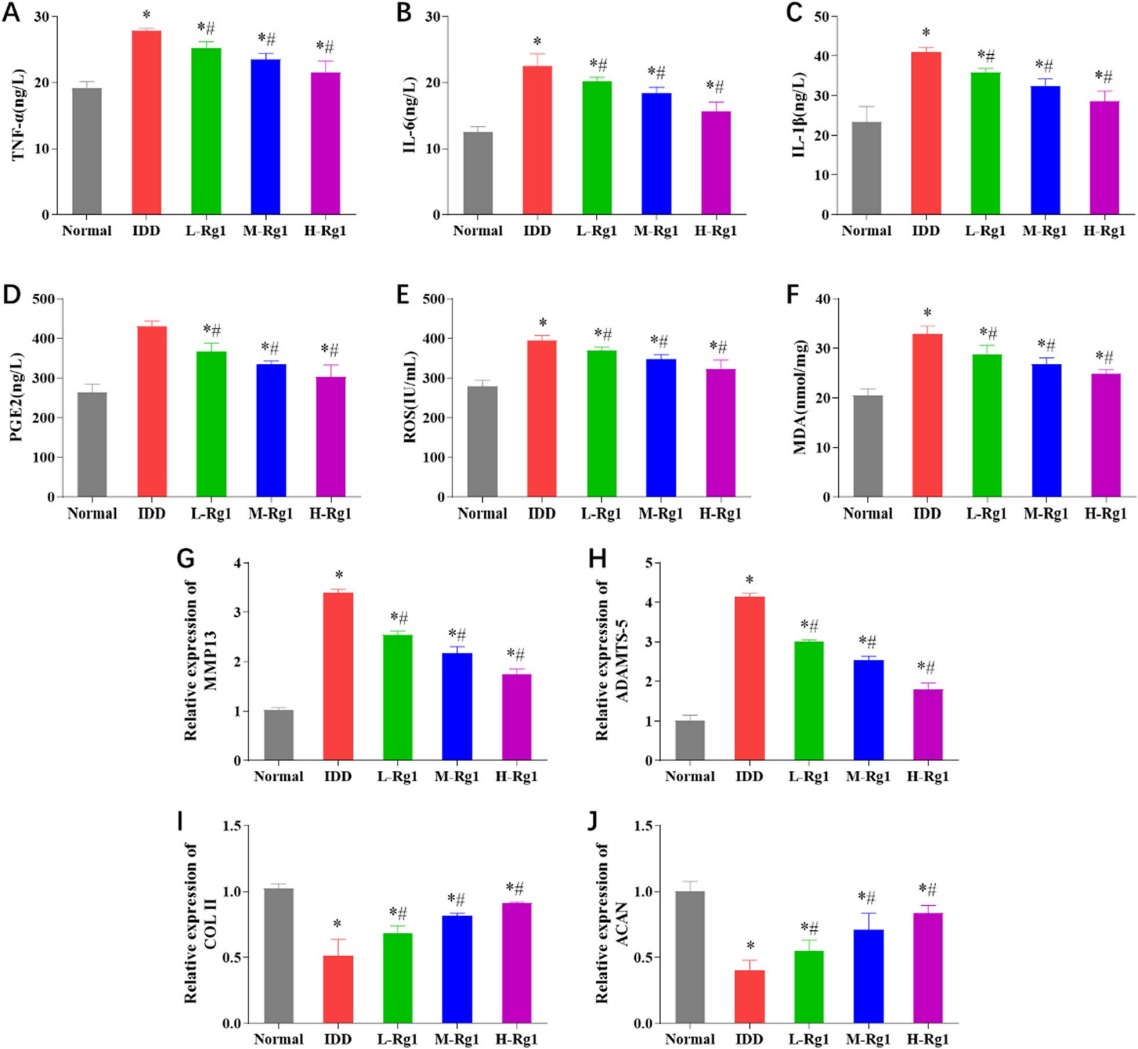
Ginsenoside Rg1 inhibits the inflammatory response, oxidative stress and extracellular matrix degradation of nucleus pulposus cells in rats with intervertebral disc degeneration. **A**–**D**: Levels of TNF-*α*, IL-6, IL-1*β*, and PGE2 in the supernatant of NP cells in each group; **E**–**F**: Activities of ROS and MDA in the supernatant of NP cells in each group; **G**–**J**: mRNA levels of ECM-related genes MMP13, ADAMTS-5, COL II, and ACAN. *p < 0.05 versus Normal group; ^#^*p* < 0.05 versus IDD group

We had measured the inflammatory levels, oxidative stress, and matrix metabolic process substances in NP tissues of IDD rats, and the results identified that ginsenoside Rg1 could inhibit the production of proinflammatory factors and oxidative stress response substances in IDD rats. Next, we carried out experiments on the cellular level. We have thus determined the secretion levels of inflammatory factors, the activity of oxidative stress response, and the gene transcription expression during ECM metabolism among in vitro NP cells; the results were consistent with the trends measured in experiments on the tissue level (Fig. 6A–J). Compared with the normal group, the IDD group presented with increases in the levels of TNF-*α*, IL-6, IL-1*β*, PGE2, ROS, MDA and ECM degradation genes, and decreases in COL II, ACAN; compared with the IDD group, the levels of TNF-*α*, IL-6, IL-1*β*, and PGE2, the activities of ROS and MDA, and ECM degradation were significantly decreased in the L-Rg1, M-Rg1, and H-Rg1 groups in a concentration-dependent manner.

### Effect of ginsenoside Rg1 on YAP1/TAZ signaling pathway in intervertebral disc tissue and nucleus pulposus cells

**Fig. 7:**
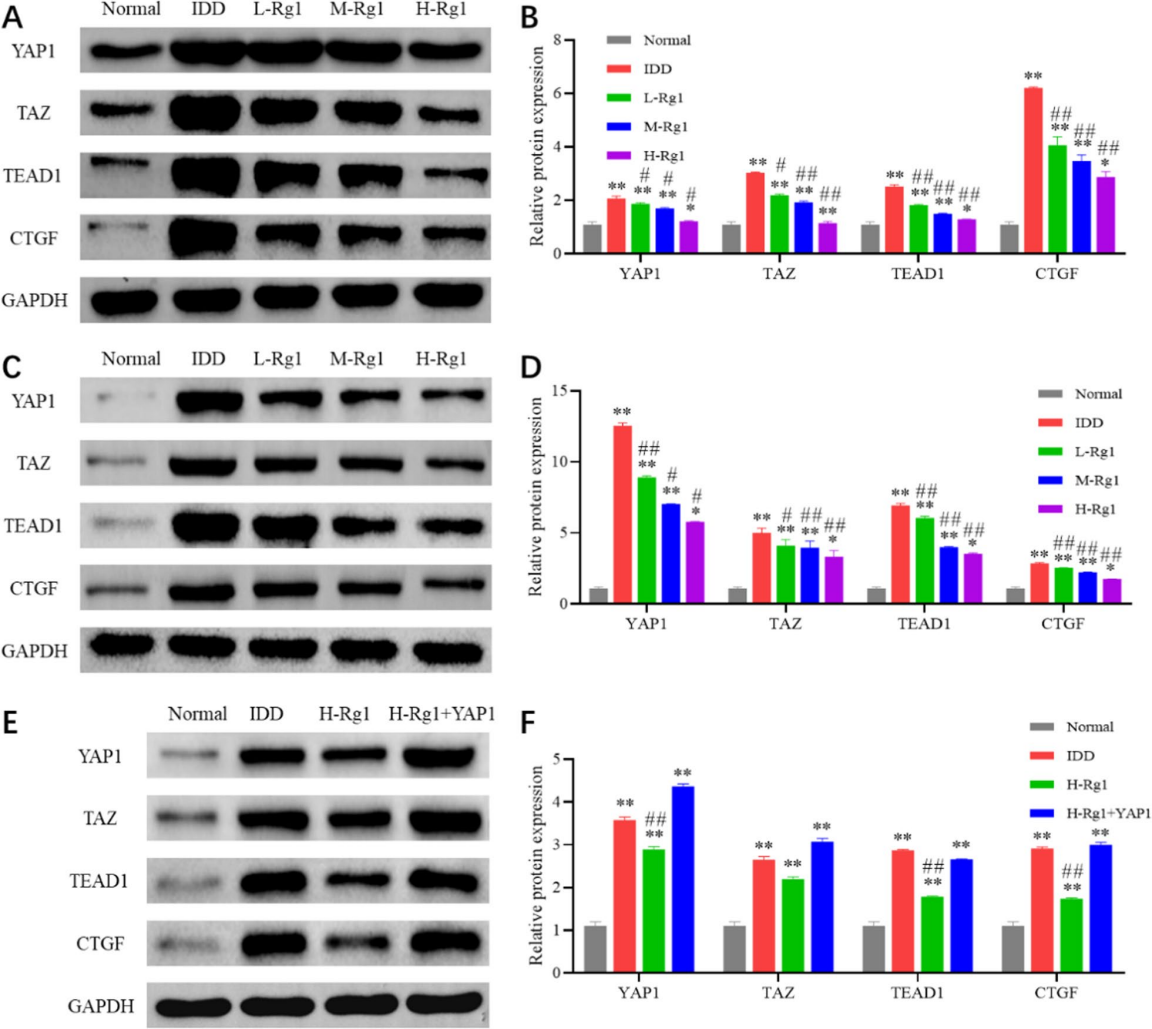
Ginsenoside Rg1 inhibits the activation of YAP1/TAZ signaling pathway in intervertebral disc tissue and nucleus pulposus cells. **A**, **B**: Expression levels of the YAP1/TAZ signaling pathway-related proteins in the IVD tissues of rats in each group were measured by western blot; **C**, **D**: Expression levels of the YAP1/TAZ signaling pathway-related proteins in NP cells of rats in each group were detected by western blot; **E**, **F**: Expression levels of YAP1/TAZ signaling pathway-related proteins in NP cells of H-Rg1 group and H-Rg1 + YAP1 group were examined by western blot. *p < 0.05,, **p < 0.01 versus Normal group; *#p* < 0.05, ^##^*p* < 0.01 versus IDD group

YAP1/TAZ plays a crucial role in NP cells and IDD progression. Our study found that IDD caused up-regulation of the expression of YAP1/TAZ pathway-related proteins YAP1, TAZ, TEAD1 and CTGF in the IVD tissue (Fig. 7A, B) and NP cells (Fig. 7C, D). By contrast, ginsenoside Rg1 contributed to the opposite effects, which significantly decreased such proteins in a concentration-dependent manner. This suggested that ginsenoside Rg1 was able to inhibit the activation of YAP1/TAZ signaling pathway in IDD rats.

To clarify the inhibitory effect of ginsenoside Rg1 on the YAP1/TAZ signaling pathway, we performed rescue experiments (Fig. 7E, F) and found that YAP1 overexpression could relieve the inhibitory effect of ginsenoside Rg1 on the YAP1/TAZ pathway, that is, the pathway was reactivated. This indicated that ginsenoside Rg1 was able to inhibit the activation of the YAP1/TAZ signaling pathway in IDD rats.

## Discussion

Currently, the clinical treatment methods for IDD include conservative and surgical treatments [20]. Common conservative therapies are bed rest, nonsteroidal anti-inflammatory drugs, and a series of common physical therapies, which cannot prevent the occurrence and development of IDD although relieving pain to some extent [21]. Surgical treatment is confronted with high cost, large trauma, and unsatisfactory postoperative rehabilitation in some patients [22]. Therefore, exploring new drugs for IDD has become an important research direction [23, 24]. Several studies have shown that ginsenoside Rg1, as an active ingredient of traditional Chinese medicine, has a wide range of pharmacological effects and high medicinal value in protecting the cardiovascular, immune, and nervous systems [25]. Our experiment presents that ginsenoside Rg1 can significantly increase mechanical and thermal thresholds in IDD rats. Safranin O-Fast green staining demonstrated that successful IDD modeling increased the histological scores, but ginsenoside Rg1 could inhibit the progression of IDD. Our study in rats suggests that ginsenoside Rg1 may also prevent and treat human IDD. However, there are still some differences between rats and human subjects, although it has been demonstrated that the morphological characteristics of histological changes of rats IVD induced by IDD modeling are almost consistent with those of human IDD patients [26]. Therefore, further experiments are needed to verify the effect of ginsenoside Rg1 in human IDD, thus providing more effective data for the development of new drugs.

The imbalance of immune response and redox can damage cells and tissues through inflammatory factors and oxidative stress substances [27], and the imbalance is closely related to histological changes in IVD and IDD development [28]. Ginsenoside Rg1 can regulate the balance of inflammatory response and oxidative stress in diabetic patients [29]. Also, our study found that ginsenoside Rg1 could reduce the inflammatory response and oxidative stress response in NP tissues of IDD rats. COL II and ACAN, as the main components of ECM, can keep the fluid within the IVD and retain the elasticity and volume of NP [30]. MMP13 and ADAMTS-5 are the main catabolic enzymes for ECM degradation [31]. A decrease in COL II, ACAN or an increase in MMP13, ADAMTS-5 may lead to an abnormality in the NP structural and ultimately result in IDD. In this study, IDD rats showed down-regulation of COL II and ACAN expression and up-regulation of MMP13 and ADAMTS-5 expression, and ginsenoside Rg1 could significantly inhibit such changes. Additionally, the apoptosis rate of NP cells was notably reduced in the IDD rats after treatment with the ginsenoside Rg1. Taken together, ginsenoside Rg1 can effectively prevent and treat IDD rats.

Accumulating evidence has highlighted that the YAP1/TAZ signaling pathway is a key regulator of bone and cartilage development, which plays a significant role in biological processes such as osteoblast differentiation, mature articular cartilage phenotype and tissue degeneration [32]. Fearing's study showed that the YAP1/TAZ signaling pathway affected NP-related cellular and phenotypic degeneration [33]. Zhang et al. reported that the activation of YAP1/TAZ signaling pathway promoted cellular senescence and IDD progression [34]. In the present study, we revealed that ginsenoside Rg1 inhibited the activation of the YAP1/TAZ signaling pathway in a concentration-dependent manner. Collectively, ginsenoside Rg1 can exert its biological function via YAP1/TAZ signaling pathway.

Existing studies have confirmed that the YAP1 signaling pathway affects IDD progression through a variety of biological processes, but our study did not further examine the phenotype of the cells in each group. Also, we did not knockdown YAP or use pathway inhibitor to examine whether ginsenoside Rg1 affects one or more phenotypes of IDD through the YAP1/TAZ signaling pathway. Additionally, it is unknown whether ginsenoside Rg1 inhibits IDD progression through other signaling pathways since we only explored one pathway. Given such lack, the mechanism of ginsenoside Rg1 inhibiting IDD progression needs to be further studied.

## Conclusion

In summary, in vitro and in vivo experiments have demonstrated that ginsenoside Rg1 can alleviate the decreased motor function and histopathological progression, inhibit inflammatory response, oxidative stress response as well as ECM degradation process in IDD rats. Such an effect of ginsenoside Rg1 on IDD progression may be achieved by inhibiting the activation of the YAP1/TAZ signaling pathway. This conclusion suggests the potential of ginsenoside Rg1 to prevent and treat IDD.

## Data Availability

The datasets are available from the corresponding authors on reasonable request.
